# Supplementary material for: Identifying barriers and enablers to rigorous conduct and reporting of preclinical laboratory studies
Source: PLoS Biol. 2023 Jan 5;21(1):e3001932. doi: 10.1371/journal.pbio.3001932 (PMC9888705; doi:10.1371/journal.pbio.3001932)
Supplement: S1 File — (PDF) [file pbio.3001932.s001.pdf]

S1_File: Description of Theoretical Domains Framework Domains

As a first step to organizing and analyzing our interview study data, using a deductive approach, we applied the Theoretical Domains Framework (TDF) to codify the data.[1] We considered 15 domains of the TDF (i.e. all 14 domains from TDF version 2 as well as the 'nature of the behavior' from TDF version 1). We have provided definitions for each domain below.

Domain	American Psychological Association (APA) Dictionary Definition[2] as originally reported by Cane et al.[3]
**Knowledge**	An awareness of the existence of something
**Skills**	An ability or proficiency acquired through practice
**Social/professional role and identity**	A coherent set of behaviours and displayed personal qualities of an individual in a social or work setting
**Beliefs about capabilities**	Acceptance of the truth, reality or validity about an ability, talent or facility that a person can put to constructive use
**Optimism**	The confidence that things will happen for the best or that desired goals will be attained
**Beliefs about Consequences**	Acceptance of the truth, reality, or validity about outcomes of a behaviour in a given situation
**Reinforcement**	Increasing the probability of a response by arranging a dependent relationship, or contingency, between the response and a given stimulus
**Intentions**	A conscious decision to perform a behaviour or a resolve to act in a certain way
**Goals**	Mental representations of outcomes or end states that an individual wants to achieve
**Memory, attention and decision processes**	The ability to retain information, focus selectively on aspects of the environment and choose between two or more alternatives
**Environmental context and resources**	Any circumstance of a person's situation or environment that discourages or encourages the development of skills and abilities, independence, social competence and adaptive behaviour
**Social influences**	Those interpersonal processes that can cause individuals to change their thoughts, feelings, or behaviours
**Emotion**	A complex reaction pattern, involving experiential, behavioural, and physiological elements, by which the individual attempts to deal with a personally significant matter or event
**Behavioural regulation**	Anything aimed at managing or changing […] actions
	**Description as per Michie et al.[4]**
**Nature of Behaviour**	Dimensions on which desired behaviours might vary

References

1. Atkins L, Francis J, Islam R, O'Connor D, Patey A, Ivers N, et al. A guide to using the Theoretical Domains Framework of behaviour change to investigate implementation problems. Implement Sci. 2017;12(1):77. PubMed PMID: 28637486; PubMed Central PMCID: PMCPMC5480145.

2. American Psychological Association. *APA Dictionary of Psychology*. (Washington, DC, 2007).

3. Cane, J., O'Connor, D. & Michie, S. Validation of the theoretical domains framework for use in behaviour change and implementation research. *Implementation Science* **7**, 37 (2012).

4. Michie, S. *et al.* Making psychological theory useful for implementing evidence based practice: a consensus approach. *Quality & Safety in Health Care* **14**, 26-33 (2005).
