# Supplementary material for: Identifying barriers and enablers to rigorous conduct and reporting of preclinical laboratory studies
Source: PLoS Biol. 2023 Jan 5;21(1):e3001932. doi: 10.1371/journal.pbio.3001932 (PMC9888705; doi:10.1371/journal.pbio.3001932)
Supplement: S2 File — (PDF) [file pbio.3001932.s002.pdf]

S2_File: Consolidated criteria for reporting qualitative studies (COREQ)[1] Checklist

No.	Item	Guide questions/description	Details (Page)
**Domain 1: Research team and reflexivity**<br>*Personal Characteristics*			
1	Interviewer/facilitator	Which author/s conducted the interview or focus group?	7
2	Credentials	What were the researcher's credentials? E.g. PhD, MD	NR (BSc Honours; MSc Epidemiology)
3	Occupation	What was their occupation at the time of the study?	7
4	Gender	Was the researcher male or female?	NR (Female)
5	Experience and training	What experience or training did the researcher have?	7
Relationship with participants			
6	Relationship established	Was a relationship established prior to study commencement?	NR (No)
7	Participant knowledge of the interviewer	What did the participants know about the researcher? e.g. personal goals, reasons for doing the research	Supplemental File 4: Interview Guide, Page 1
8	Interviewer characteristics	What characteristics were reported about the interviewer/facilitator? e.g. Bias, assumptions, reasons and interests in the research topic	7
**Domain 2: Study design**<br>*Theoretical framework*			
9	Methodological orientation and Theory	What methodological orientation was stated to underpin the study? e.g. grounded theory, discourse analysis, ethnography, phenomenology, content analysis	6-8
Participant selection			
10	Sampling	How were participants selected? *e.g. purposive, convenience, consecutive, snowball*	7
11	Method of approach	How were participants approached? *e.g. face-to-face, telephone, mail, email*	7
12	Sample size	How many participants were in the study?	9
13	Non-participation	How many people refused to participate or dropped out? Reasons?	NR
Setting			
14	Setting of data collection	Where was the data collected? e.g. home, clinic, workplace	7 (phone)<br>NR (in person: office/ conference room)
15	Presence of non-participants	Was anyone else present besides the participants and researchers?	N/A
16	Description of sample	What are the important characteristics of the sample? *e.g. demographic data, date*	9, Table 1

Data collection			
17	Interview guide	Were questions, prompts, guides provided by the authors? Was it pilot tested?	6-7<br>Supplemental File 4: Interview Guide<br>page 3, 8
18	Repeat interviews	Were repeat interviews carried out? If yes, how many?	N/A
19	Audio/visual recording	Did the research use audio or visual recording to collect the data?	7
20	Field notes	Were field notes made during and/or after the interview or focus group?	N/A
21	Duration	What was the duration of the interviews or focus group?	9, Table 1
22	Data saturation	Was data saturation discussed?	NR
23	Transcripts returned	Were transcripts returned to participants for comment and/or correction?	N/A
**Domain 3: Analysis and findings**<br>*Data analysis*			
24	Number of data coders	How many data coders coded the data?	7-8
25	Description of the coding tree	Did authors provide a description of the coding tree?	7-8, Supplemental File 1
26	Derivation of themes	Were themes identified in advance or derived from the data?	7-8
27	Software	What software, if applicable, was used to manage the data?	NR (NVivo, Excel)
28	Participant checking	Did participants provide feedback on the findings?	N/A
Reporting			
29	Quotations presented	Were participant quotations presented to illustrate the themes / findings? Was each quotation identified? e.g. participant number	10-17
30	Data and findings consistent	Was there consistency between the data presented and the findings?	9-17 and Supplemental File 5
31	Clarity of major themes	Were major themes clearly presented in the findings?	9-16
32	Clarity of minor themes	Is there a description of diverse cases or discussion of minor themes?	9-17 and Supplemental File 5

References

1. Tong, A., Sainabury, P. & Craig, J. Consolidated criteria for reporting qualitative research (COREQ): a 32-item checklist for interviews and focus groups. International Journal for Quality in Health Care 19, 349–357.
