# Supplementary material for: Identifying barriers and enablers to rigorous conduct and reporting of preclinical laboratory studies
Source: PLoS Biol. 2023 Jan 5;21(1):e3001932. doi: 10.1371/journal.pbio.3001932 (PMC9888705; doi:10.1371/journal.pbio.3001932)
Supplement: S3 File — (PDF) [file pbio.3001932.s003.pdf]

S3_File: Definition of behaviour using the Action, Actor, Context, Target, Time (AACTT) framework[1]

**Action**	Implementation of the seven NIH principles (Use of community-agreed standards in reporting, Distinguishing biological and technical replicates, Statistics, Randomization, Blinding, Sample size estimation, Inclusion and exclusion criteria)
**Actor**	Preclinical researchers conducting and reporting *in vivo* experiments
**Context**	Laboratory and/or office
**Target**	Preclinical researchers/ research community who read and use results from preclinical *in vivo* studies
**Time**	During design, conduct and reporting of preclinical *in vivo* experiments

Reference

1. Presseau J, McCleary N, Lorencatto F, Patey AM, Grimshaw JM, Francis JJ. Action, actor, context, target, time (AACTT): a framework for specifying behaviour. Implement Sci. 2019 Dec 5;14(1):102. doi: 10.1186/s13012-019-0951-x. PMID: 31806037; PMCID: PMC6896730.
