# Supplementary material for: Identifying barriers and enablers to rigorous conduct and reporting of preclinical laboratory studies
Source: PLoS Biol. 2023 Jan 5;21(1):e3001932. doi: 10.1371/journal.pbio.3001932 (PMC9888705; doi:10.1371/journal.pbio.3001932)
Supplement: S4 File — (PDF) [file pbio.3001932.s004.pdf]

S4_File: Interview Guide for Researchers

<u>**Note:** Headers throughout the main interview questions are meant to demonstrate which domain the questions are intended to address, along with related theoretical constructs associated with each domain. These were meant to aid research assistants when conducting and analyzing the interviews.</u>

Explanation *(read to researchers)*

Thank you for agreeing to speak with me today about your knowledge and perspective on guidelines regarding experimental design and reporting. By conducting interviews with preclinical scientists we hope to better inform the development of a workshop and online platform, to ensure these tools will be useful to preclinical scientists. The interview should take approximately 45 minutes and will be audio-recorded to ensure that all key points are accurately documented.

It is important to note that there are no right or wrong answers to the questions; rather, we are interested in what you know and your views about experimental design and reporting. We're really hoping to get your honest answers. We realize that it may not always be possible to apply all design and reporting guidelines, and that's why we're interested in getting your perspective. We're hoping to better understand current practices and potential enablers and barriers in the preclinical setting so that these can be incorporated and addressed in the workshop and online platform.

I have no vested interest in your answers, and your responses will be effectively anonymous. As soon as we finish, I'll assign your audio file a unique ID code and store it on the hospital server. The only people who will listen to the audio file will be authorized study personnel. When the audio file is converted to text format we will ensure that any and all identifying information (for example, your name or names of other individuals that you use in the course of our discussion) is removed from the interview transcripts, making you effectively anonymous.

If you wish to end the interview before I have asked all of the questions or if you wish to withdraw from the study you are free to do so.

Also, just a note, there may appear to be some overlap between questions (some questions may seem repetitive). However, each question has been worded in a certain way to obtain specific information.

Any questions before we start?

Domain Knowledge (Construct = *Knowledge*):

1. Can you tell me about any guidelines concerning the designing and reporting of *in vivo* preclinical studies that you are aware of?
2. Have you previously heard of the NIH's Principles and Guidelines for Reporting Preclinical Research?

Background *(read to researchers)*

Just so that we are on the same page I'm going to provide you with a brief background. These principles and guidelines were created in a joint workshop held between NIH and the journal Nature.[1] Journal editors who attended the workshop came to a consensus and established a core set of principles and guidelines for reporting study design.

Journals and authors can use these principles and guidelines as a checklist during the editing process of their manuscript to identify whether or not the information is present. Scientists can also use these guidelines when designing a study. These guidelines encompass 7 core domains.

For consistency I'm just going to give you a standard definition[1] that I read for all of the interviews, so that everyone is on the same page. **Standards**: refers to the use of community-agreed standards in reporting research in peer reviewed publications. Examples include the use of the Animal Research: Reporting of In Vivo Experiments (ARRIVE) guidelines,[2] or the Nature Publishing Group reporting checklist.[3] These are checklists of suggested items to report in a manuscript.

Replicates: This domain refers to reporting how often the experiment was performed and whether the results were substantiated by repetition under a range of conditions.

Additionally, guidance suggests distinguishing between biological (i.e. n=6 mice) and technical replicates (i.e. n=6 measurements on 1 mouse). So for instance, when reporting a figure in a manuscript, ensuring that it is clear whether data points represent biological or technical replicates.

Definitions (Don't need to read, only if they ask for clarification):

- *Biological replicates: "Biological replicates are parallel measurements of biologically distinct samples that capture random biological variation, which may itself be a subject of study or a source of noise."*[4]
- *Technical replicates: "Technical replicates are repeated measurements of the same sample that represent independent measures of the random noise associated with protocols or equipment."*[4]

Statistics: Suggests full reporting of the statistical tests used, exact value of N, and definition of center, dispersion and precision measures (e.g. mean/median, SEM/SD), etc.

Randomization: Report whether or not samples were randomized and specify the method of randomization (i.e. computer-produced random sequence[5]).

Blinding: Report whether researchers were blinded to group assignment a) during the experiment and b) during outcome assessment/analysis.

Sample-size estimation: State whether sample size was calculated during study design phase and report the method of computation.

Inclusion and exclusion criteria: Define the criteria used to include or exclude any data or subjects (animals). Describe any outcomes that were measured but not reported in the results section.

In relation to that, for the purposes of this interview, I would like you to think of the following specific activity:

You personally **applying the methods outlined by the NIH Principles and Guidelines[1] to A) design and B) report** an *in vivo* preclinical study.

A) Applying these methods during the study design phase would include:

- Using community based standards to help report the study
- Determining whether biological replicates or technical replicates will be used
- Determining the details of statistical analysis, randomization and blinding
- Calculating the sample size
- Determining any *a priori* inclusion/exclusion criteria

3. **Open Question:** Do you foresee any barriers to applying the study design guideline methods? (Can you think of any reasons why you may not be able to apply them? Have you faced any barriers?)

Domain Skills (Construct = Skills):

4. *Skills*: Have you received training on designing a study previously?
    - PROMPT: IF so, what did the training consist of?
    - PROMPT: Is there anything you would want (further) training on?

Domain Social/Professional Role and Identity (Construct = *Professional Role*):

5. *Professional Role*: What is your role in the experimental design of a study, in terms of each NIH domain?
    - Prompt (*Ensure **all** prompts are asked): randomization? Blinding? Inclusion/exclusion criteria? Reporting standards? Statistics? Replicates?
    - Prompt: what about colleagues, principal investigators, research team
    - PROMPT: Who do you think is responsible for applying these methods? Anyone else?

Here's a sheet that you can refer to throughout the interview as the remaining questions will focus on the use of these principles and guidelines.

6. When you think about what preclinical scientists "do", how/where do you see these guidelines fitting into how you see yourself as a preclinical researcher?

Domain Beliefs About Capabilities (Constructs = *Self-efficacy* and *Perceived Behavioural Control*)

7. *Self-efficacy:* How confident are you that you can apply all of the experimental design guideline methods?
    - PROMPT: What are some factors that might make you feel less confident that you can apply the experimental design guideline methods for a study?
    - PROMPT: And what factors make you feel more confident
8. *Perceived Behavioural Control:* What makes it easier for you to apply the experimental design guideline methods?
    - PROMPT: What makes it more difficult or impossible to apply the experimental design guideline methods?

Domain Optimism (Constructs = *Optimism* and *Pessimism*)

9. *Optimism:* Do you expect that applying the experimental design guideline methods will result in more good things than bad?
    - PROMPT *(if interviewee does not understand):* Do you expect that applying the experimental design guideline methods will be beneficial, or challenging? More positive or negative outcomes?
    - PROMPT: Why is that? Can you give me an example?

Domain Beliefs About Consequences (Constructs = *Attitudes* and *Outcome Expectancies*):

10. *Outcome Expectancies:* What are some of the benefits of applying the experimental design guideline methods for a study?
    - PROMPT: for yourself?
    - PROMPT: for your fellow colleagues on the same study?
    - PROMPT: for the field of preclinical research?
    - PROMPT: And what about eventually for patients? (If they do not understand the prompt: if these methods help to improve reproducibility, and help to identify the most promising therapies)?
11. *Outcome Expectancies:* What do you anticipate are some of the disadvantages of applying the experimental design guideline methods?
    - Same prompts as above

Domain Reinforcement (Construct = Reinforcement):

12. *Reinforcement*: What would motivate you to apply the experimental design guideline methods?
    - PROMPT: Financial incentives?
    - PROMPT: Recognition?
    - PROMPT: Unable to publish in certain journals?
    - PROMPT: Unsuccessful in grant applications?

Domain Intentions (Construct = Intentions):

13. *Intentions:* If you were to start a new study in the next 2 months, do you intend to apply the experimental design guideline methods throughout the duration of your study?
    - Prompt: Why or why not?

14. *Intentions:* How motivated would you be to apply the experimental design guidelines if you knew this could reduce the magnitude of effect seen in your experiments?
    a. PROMPT: Why is that??

Domain Goals (Construct = *Priority*):

15. *Priority:* Generally, how much of a priority is applying the guidelines during the design phase?
    - Prompt: As compared to having deliverables? Getting expected results?
    - PROMPT: Submitting grants, presenting results (conferences), managing staff, teaching, etc.
    - How important are each of the domains (equally important or are some more of a priority than others)?

Domain Memory, Attention and Decision Processes (Constructs = *Memory* and *Attention*)

16. *Memory:* Can you give me an idea of situations or types of experiments, where you may be more likely to forget to apply the guideline methods during experimental design?
17. *Attention:* Can you tell me about any competing demands that may interfere with you ensuring that all experimental design guidelines are applied?

Domain Environmental Context and Resources (Constructs = Resources Material):

18. *Resources Material:* What resources at your center do you feel (would) facilitate your application of the experimental design guideline methods?
19. *Resources Material:* What resources do you need to be made available to you in order to apply the methods in the experimental design guidelines?
    - Prompt: Financial?
    - Prompt: Institutional support (hospital), or lab/team assistance?
    - Prompt: Additional information?
    - Prompt: Logistical challenges?
    - Can any of these be improved?

Domain Social Influences (Constructs = Social Support, Subjective Norm, and Descriptive Norm):

20. *Social Support:* Whose opinion do you take into consideration to decide whether to apply experimental design guideline methods?

- Prompt: Senior staff? Fellow lab mates? Institutional guidelines?

21. *Social Support:* Whose support helps you apply the experimental design guideline methods?
    - Prompt: Senior staff? Fellow lab mates? Institutional guidelines?

Domain Emotion (Constructs = Affect and Stress):

22. *Affect*: When thinking about applying the design guideline methods, what emotions come to mind?
    - PROMPT: (prompt key potential emotions: guilt, worry, concern, satisfaction, inspired, nervous, stressed, happy, sad, urgency)
    - PROMPT: Tense Situation= An experiment doesn't have the expected results, something goes wrong with the experiment, competing priorities and deadlines (grant applications, conferences, etc.)

Domain Behavioural Regulation (Constructs = Automaticity, Self-Monitoring, and Action Planning):

23. *Habit:* What needs to happen for application of the design guideline methods to become routine?
24. Based on your experiences, do you have any suggestions, thoughts or **strategies** you would recommend as to how yourself and others can apply experimental design guideline methods?

25. Thinking about everything we've just discussed, in your opinion, what are the most important factors that would influence whether you would apply the experimental design methods outlined in the NIH guidelines?

Other questions

1. Would you attend an in-person workshop and/or online platform of educational resources for experimental design and reporting if one is available in the next year?
2. *Environmental Context & Resources/ Knowledge:* What tools or information do you feel would best inform you on experimental design methods?
    - PROMPT: Brochure, website, smart phone app, meeting with the lab team?

Is there anything else you wanted to say or expand on about?

Background of Informant

And I just have a few final questions so that we can describe our sample of interviewees:

1. Male or Female (to keep track of, won't be asked)
2. Would you be comfortable telling me your age or what year you were born in?
3. What is the title of your current position?

4. How many years have you been a cancer/cardiovascular researcher (including graduate studies)?

Thank-you for participating.

References

1. National Institutes of Health. Principles and Guidelines for Reporting Preclinical Research. https://www.nih.gov/research-training/rigor-reproducibility/principles- guidelines-reporting-preclinical-research. Accessed February 11 2022.

2. Kilkenny C, Browne WJ, Cuthill IC, Emerson M, Altman DG. Improving Bioscience Research Reporting: The ARRIVE Guidelines for Reporting Animal Research. PLoS Biol. 2010;8(6):e1000412. doi:10.3390/ani4010035.
3. Henderson V, Kimmelman J, Fergusson D, Grimshaw J, Hackam D. Threats to Validity in the Design and Conduct of Preclinical Efficacy Studies: A Systematic Review of Guidelines for In Vivo Animal Experiments. PLoS Med. 2013;10(7):e1001489. doi:10.1371/journal.pmed.1001489.
4. National Institutes of Health. NIH Rigor and Reproducibility Training Module 3: Biological and Technical Replicates. https://www.nigms.nih.gov/training/documents/module3-biological-and-technical-replicates.pdf. Accessed December 22, 2017.
5. Higgins J, Altman D, Sterne J (editors). Chapter 8: Assessing risk of bias in included studies. In: Higgins J, Green S, eds. Cochrane Handbook for Systematic Reviews of Interventions. Version 5. 1.0 (updated March 2011). The Cochrane Collaboration; 2011. Available from www.handbook.cochrane.org.

NIH Principles and Guidelines for Reporting Preclinical Research

**Standards**	• Use of community-agreed standards in reporting
**Replicates**	• Reporting how often the experiment was performed and whether the results were substantiated by repetition under a range of conditions<br>• Distinguishing between biological and technical replicates
**Statistics**	• Full reporting of the statistical tests used, exact value of N, and definition of center, dispersion and precision measures
**Randomization**	• Reporting whether or not the samples were randomized and specifying the method
**Blinding**	• Reporting whether or not researchers were blind to group assignment *during the experiment* and *during outcome assessment*
**Sample size estimation**	• Stating whether or not sample size was calculated and reporting the method
**Inclusion and exclusion criteria**	• Defining the criteria used to include or exclude any data or subjects<br>• Describing any outcomes that were measured but not reported in the results

For the purposes of this interview, please think of the following specific activity:

You personally **applying the methods outlined by the NIH Principles and Guidelines to A) design and B) report** an *in vivo* preclinical study.
