# Supplementary material for: Identifying barriers and enablers to rigorous conduct and reporting of preclinical laboratory studies
Source: PLoS Biol. 2023 Jan 5;21(1):e3001932. doi: 10.1371/journal.pbio.3001932 (PMC9888705; doi:10.1371/journal.pbio.3001932)
Supplement: S5 File — (PDF) [file pbio.3001932.s005.pdf]

S5_File: Sub-themes, Belief Statements and Frequencies

Table 1) Barrier Theme 1: There is variability in awareness and current practices

Sub-Theme	Belief Statements		Frequency	TDF Domains
Varied awareness of specific guidelines	I ***have*** previously heard of the NIH's Principles and Guidelines for Reporting Preclinical Research		21	Knowledge, Skills, Nature of behaviour
	I'm not sure if I have previously heard of the NIH's Principles and Guidelines		1	
	I ***have not*** previously heard of the NIH's Principles and Guidelines (or not before this study)		8	
	My knowledge of the NIH's Principles and Guidelines for Reporting Preclinical Research is limited		5	
	Aware of other guidelines concerning the designing and reporting of in vivo preclinical studies		13	
	I am aware of other guidelines (but am not sure of the specific name)		4	
	I am not aware of any official guidelines, but am aware of some important design elements		3	
	I was not aware of any formal guidelines (or not before this study)		4	
	I am not aware of guidelines but am aware of statements addressing the need for such guidelines		1	
Varied training	Have you ever received training on designing a study previously?	Yes (school, workshop, consult with research services/biostatistician)	10	
		No, just informal training through school and mentoring in the lab	8	
		For clinical research (I apply to preclinical research)	2	
		Self-taught (I am a professor)	2	
		No, but I think my students have	1	
		No (nothing formal or official)	9	
	Training is important/ would be helpful for others (e.g. students, personnel)		14	
	I would like further training (e.g. sample size estimation, statistics, blinding, etc.)		9	
	I would not want further training in this area/ not a priority for me		2	
Varied views on whether implementation is an issue	Aware of gaps in implementing the NIH core domains (e.g. sample size estimation, statistics)		10	
	Aware of gaps in reporting of preclinical research		4	
	I think there was a gap, but now everyone applies the guidelines because of journal requirements		1	
	Guidelines articulate what we (and our colleagues) were already doing		2	
	Think most labs are applying some of the domains (e.g. randomization)		2	
	Didn't know these practices weren't routine; believe the issue lies in journal space limitations		2	
Disconnect between stages of implementation and guidelines	Others may not (or I did not) realize there were reporting guidelines were until submitting to the journal		3	

Table 1) Barrier Theme 1: There is variability in awareness and current practices (Continued)

Sub-Theme	Belief Statements		Frequency	TDF Domains
Varied current practices	Overall	Have prior experience (already follow most or all of the guidelines)	17	Knowledge, Skills, Nature of behaviour
		Apply most of these domains (but don't formally go through checklist)	5	
	Blinding	Have prior experience (or team does)	23	
		Do not currently implement	5	
	Randomization	Have prior experience (or team does)	21	
		Do not currently implement	2	
	Sample Size Estimation	Have prior experience (or team does)	11	
		Sometimes use a certain number of mice (e.g. to start then can replicate, based on literature, generally accepted, physically possible, power of positive results)	7	
		Do not currently implement	6	
	Statistics	Have prior experience (or team/ statistician does) in conducting or reporting statistics	22	
		Do not determine statistical tests beforehand	1	
	Inclusion & Exclusion Criteria	Have prior experience (or our team does)	15	
		No formal criteria, could improve	5	
		Exclusion decisions usually post-experiment	5	
		Don't necessarily report all exclusions (e.g. data not acquired- technical or health issues)	3	
	Replicates	Have prior experience (or team does) in determining or reporting replicates	21	
		Do not usually repeat under a range of conditions (but do repeat, or try another model)	2	
		Do not usually replicate same experiment (but do replicate in another model)	1	
		Do not always report (e.g. number of experiments, distinguish biological vs. technical)	3	
	Community agreed standards in reporting	Have prior experience (or team does)	6	
		Have not yet implemented (usually look to other papers)	4	
Potential misconceptions/ areas for improvement	Do not use software or a formal approach to randomize		4	
	Randomize by cage		2	
	Not standard to report certain details (e.g. sample size)		3	
	Sample size estimation may have a bigger role in clinical studies		2	
	Statistics during study design phase is not a priority, can always change later		1	
	Not much weight to randomization in animals (in-bred mice)		1	
	Inclusion and exclusion criteria not as important as we are all using the same strain of rats		1	
	Challenges with blinding; someone must know what the groups are		1	
	Sample size calculation is irrelevant by the time you are reporting on the experiment		1	

Table 2) Barrier Theme 2: Costs and challenges of implementing the guidelines

Sub-Theme	Belief Statements	Frequency	TDF Domains
Negative impact on resources	Financial costs associated with implementing the design guideline methods	20	Beliefs about consequences, Beliefs about capabilities, Environmental context and resources, Goals
	Sample size might be very large	19	
	Takes more time to apply the guidelines (might slow down your experiments)	10	
	Increases number of people involved/ needing to synchronize	8	
	Certain aspects may be more work (tedious, more planning, organization)	9	
Resources required	Cost implications are a barrier	17	
	Need financial resources to implement	16	
	Sometimes it is not financially feasible to implement the guidelines	7	
	Finances (cost, financial feasibility, grants) will influence my implementation of the guidelines	6	
	Funding may be a barrier to some labs (labs with a new investigator, not well funded)	5	
	Needing additional personnel to help implement is a barrier (prohibitive to small/ new labs, trainees working on projects alone)	19	
	It will (or does) take time to implement the guidelines	13	
	Time may be a barrier	12	
	Size of experiment may be a barrier	13	
Not (always) feasible	There are difficulties to implementing the guidelines, sometimes not feasible (e.g. cost, discovery research, working by yourself)	15	
	Estimating sample size can be difficult or challenging (e.g. primary outcome not defined, not sure of effect size, not physically or financially possible)	17	
	Blinding can be difficult or challenging (e.g. personnel, cannot blind some models)	15	
	Randomization can be difficult or challenging (e.g. cost, personnel)	9	
	Not feasible to implement the guidelines ***all*** the time (guidelines, need to be flexible)	5	
Aim of research	The aim of the experiment (confirmatory versus exploratory) influences my application of the experimental design guideline methods	15	

Table 3) Enabler Theme 1: Benefits and proficiency increase motivation and intentions

Sub-Theme	Belief Statements	Frequency	TDF Domains
Positive beliefs about consequences	Improves robustness, quality, accuracy reliability of the data; will help to get 'real' answer	21	Beliefs about consequences, Goals, Beliefs about Capabilities, Nature of behaviour, Intention
	Hopefully improve reproducibility or make replication easier; want work to be reproducible	17	
	Applying the experimental design guidelines will result in more good things than bad	18	
	More confidence and trust in data	11	
	Reduce or minimize bias	9	
	May improve translation or is important to ensure appropriate discoveries selected for translation (facilitate, more stringent, more solid evidence base)	8	
Motivating factors	It's 'good science' (want results to be reliable/reproducible, to be rigorous, want to benefit the scientific community, want to determine the 'true' effect)	22	
	Ability to publish (especially in high impact/reputable journals) and journal or grant requirements would be motivating	12	
	For translation to clinical research or treatments	5	
Feasible practices	I do not see many barriers to implementing the guidelines, most are feasible	11	
	Blinding is feasible (e.g. animal technician helps us)	8	
	Randomization is feasible (e.g. standard, done by our animal tech)	4	
	Estimating sample size seems/is feasible (e.g. work with a statistician, just need to learn)	4	
Confident in ability to implement	How confident are you that you can apply all of the experimental design guideline methods?<br>• Very confident<br>• Confident/ Fairly confident<br>• Confident about implementing some domains, but not others<br>• Not entirely confident	 6<br>13<br>5<br>2	
High intention	If you were to start a new study in the next two months, do you intend to apply the experimental design guideline methods throughout the duration of your study?<br>• Yes<br>• Yes, we already apply implement some or most of the guidelines<br>• Yes, but not all domains<br>• Probably not, will take more time	 17<br>10<br>2<br>1	
	Would you attend an in-person workshop and/or online platform of educational resources?<br>• Yes/probably<br>• I would send my students or trainees<br>• Depends on schedule, length and timing<br>• More likely if online<br>• No/ probably not	 22<br>5<br>3<br>2<br>3	

Table 4) Enabler Theme 2: Need for support, resources and system level changes

Sub-Theme	Belief Statements	Frequency	TDF Domains
Education and training	General education or training (e.g. for everyone) would be helpful	27	Environmental Context and Resources, Behavioural Regulation, Reinforcement, Social Influences, Social Professional Role & Identity
	Helpful to learn early or at start of training	12	
Personnel	We involve others or work in teams (e.g. personnel, facility techs, collaborators)	15	
	Our animal care personnel are helpful	9	
	Designated person to help train/implement/provide support would be helpful	9	
	More personnel to implement would (or could) be helpful	7	
Support from specialized personnel	We consult with a biostatistician (sometimes/have before)	15	
	We have a help center/department or a consult service (for a fee)	8	
	We have access to biostatistics help, but have not used it	3	
	Access to a biostatistician or statistical resources would be helpful	6	
	Others may benefit from access to a biostatistician	2	
	Working with a biostatistician is helpful	2	
Social Support	Who's support helps you apply the guideline methods?<br>• Lab team or staff<br>• Principal Investigator<br>• Students and trainees<br>• Technician/ Animal Technician<br>• Animal Care<br>• More senior staff (e.g. lab supervisor or manager)<br>• No one<br>• Funder/ Grant Agency<br>• Institutional guidelines	 11<br>8<br>7<br>6<br>5<br>3<br>3<br>2<br>1	
Financial resources	More financial resources would be helpful	6	
	More financial resources would be helpful (but I do have access)	3	
	We have the financial resources to do this (makes it easier)	2	
	Funding is not a problem for me currently	1	
Harmonized requirements	Grant /journal requirements will encourage implementation and be helpful	14	
	Need to harmonize/formalize guidelines or training (e.g. journals, institution)	10	
	Guidelines not currently harmonized or formal (e.g. across journals, labs)	5	
	Animal care or biohazard guidelines may be a barrier, need to balance	5	
	Journal space restrictions and limits may be a barrier	5	
	Requirements do/will influence my application of the guidelines	3	
