# Supplementary material for: Identifying barriers and enablers to rigorous conduct and reporting of preclinical laboratory studies
Source: PLoS Biol. 2023 Jan 5;21(1):e3001932. doi: 10.1371/journal.pbio.3001932 (PMC9888705; doi:10.1371/journal.pbio.3001932)
Supplement: S6 File — (PDF) [file pbio.3001932.s006.pdf]

S6_File: Detailed Description of Mapping Exercise Methods and Results

a) Detailed description of mapping exercise methods to identify potentially relevant implementation strategies

To identify possible implementation strategies for preclinical researchers, relevant TDF domains from the interview study were mapped to the Behaviour Wheel of Change (BCW; Supplemental File 7).[1, 2] This framework provides a comprehensive overview of intervention functions and policies, linked to conditions for behaviour (capability, opportunity and motivation, or the 'COM-B' system).[2] The TDF domains have also been mapped to the COM-B system, allowing for the identification of possible intervention functions and policies according to relevant domains.[1] We then identified potentially relevant implementation strategies from the Expert Recommendations for Implementing Change (ERIC) project[3] for preclinical researchers (e.g. specific actions that can be taken by these individuals to improve implementation of the NIH Principles and Guidelines). ERIC strategies were coded by one researcher (MF) for a) relevance/applicability to investigators and Highly Qualified Personnel (HQP; e.g. Research Associates, Post-Doctoral Fellows, PhD students); and b) most appropriate BCW intervention function and/or policy category classification. Two independent researchers (MJ, AS) audited these results, first confirming coding to the BCW intervention functions and policies, and then confirming relevance/applicability to investigators and HQP. Conflicts were resolved through consensus discussions with all three researchers, and consultation with a fourth reviewer (ML or DAF) as needed.

b) Detailed description of mapping exercise results

In the main text, Table 2 and Figure 1 describe proposed intervention functions, policies, and implementation strategies to address identified barriers. Mapping of relevant TDF domains to the Behaviour Change Wheel [1, 2], identified 16 potentially relevant intervention function and policy categories (see Supplemental File 7 for additional details). Fifty of the 73 ERIC implementation strategies [3] mapped to 10 of these relevant intervention function and policy categories (Education, Communication/Marketing, Persuasion, Training, Environmental/Social Planning, Modelling, Environmental Restructuring, Service Provision, Guidelines, Enablement) and were relevant to preclinical researchers. A condensed summary of the results has been presented in the main text, full mapping and coding results can be found in Supplemental File 7, 8 and 9.

References

1. Atkins L, Francis J, Islam R, O'Connor D, Patey A, Ivers N, et al. A guide to using the Theoretical Domains Framework of behaviour change to investigate implementation problems. Implement Sci. 2017;12(1):77. PubMed PMID: 28637486; PubMed Central PMCID: PMCPMC5480145.
2. Michie S, van Stralen MM, West R. The behaviour change wheel: a new method for characterising and designing behaviour change interventions. Implement Sci. 2011;6:42. Epub 2011/04/26. doi: 10.1186/1748-5908-6-42. PubMed PMID: 21513547; PubMed Central PMCID: PMCPMC3096582.
3. Powell BJ, Waltz TJ, Chinman MJ, Damschroder LJ, Smith JL, Matthieu MM, et al. A refined compilation of implementation strategies: results from the Expert Recommendations for Implementing Change (ERIC) project. Implement Sci. 2015;10:21. Epub 2015/04/19. doi: 10.1186/s13012-015-0209-1. PubMed PMID: 25889199; PubMed Central PMCID: PMCPMC4328074.
