# Supplementary material for: Identifying barriers and enablers to rigorous conduct and reporting of preclinical laboratory studies
Source: PLoS Biol. 2023 Jan 5;21(1):e3001932. doi: 10.1371/journal.pbio.3001932 (PMC9888705; doi:10.1371/journal.pbio.3001932)
Supplement: S7 File — (PDF) [file pbio.3001932.s007.pdf]

S7_File: Mapping of TDF Domains to the Behaviour Change Wheel Intervention Functions and Policies[1,2]

Table 1) Mapping Results

Theme	TDF Domain	COM-B System	Intervention Functions	Policies
Barrier Theme 1: There is variability in awareness and current practices	Knowledge	Capability - Psychological	Education, Training, Enablement	• Communication/Marketing<br>• Guidelines<br>• Fiscal<br>• Regulation<br>• Legislation<br>• Environmental/social planning<br>• Service Provision
	Skills (cognitive)			
	Nature of Behaviour			
Barrier Theme 2: Costs and challenges of implementing the guidelines	Beliefs about Consequences	Motivation - Reflective	Education, Persuasion, Incentivization, Coercion	• Communication/Marketing<br>• Guidelines<br>• Fiscal<br>• Regulation<br>• Legislation<br>• Service Provision
	Beliefs about Capabilities			
	Goals			
	Environmental Context and Resources	Opportunity - Physical	Restriction, Environmental Restructuring, Enablement	• Guidelines<br>• Fiscal<br>• Regulation<br>• Legislation<br>• Environmental/social planning<br>• Service Provision
Enabler Theme 1: Benefits and proficiency increase motivation and intentions	Beliefs about Consequences	Motivation - Reflective	Education, Persuasion, Incentivization, Coercion	• Communication/Marketing<br>• Guidelines<br>• Fiscal<br>• Regulation<br>• Legislation<br>• Service Provision
	Beliefs about Capabilities			
	Goals			
	Intention			
	Nature of Behaviour			

Note: As the BCW was mapped to TDF Version 2, the 'Nature of Behaviour' domain is not included. ***Abbreviations:*** Behaviour Change Wheel (BCW); Capability, Opportunity, Motivation – Behaviour (COM-B); Theoretical Domains Framework (TDF).

Table 1) Mapping Results (*Continued*)

Theme	TDF Domain	COM-B System	Intervention Functions	Policies
Enabler Theme 2: Need for support, resources and system level changes	Environmental Context and Resources	Opportunity - Physical	Restriction, Environmental Restructuring, Enablement	• Guidelines<br>• Fiscal<br>• Regulation<br>• Legislation<br>• Environmental/social planning<br>• Service Provision
	Behavioural Regulation	Capability - Psychological	Education, Training, Enablement	• Communication/Marketing<br>• Guidelines<br>• Fiscal<br>• Regulation<br>• Legislation<br>• Environmental/social planning<br>• Service Provision
	Reinforcement	Motivation – Automatic	Persuasion, Incentivization, Coercion, Environmental restructuring, Modelling, Enablement	• Communication/Marketing<br>• Guidelines<br>• Fiscal<br>• Regulation<br>• Legislation<br>• Environmental/social planning<br>• Service Provision
	Social Influences	Opportunity – Social	Restriction, Environmental Restructuring, Enablement	• Guidelines<br>• Fiscal<br>• Regulation<br>• Legislation<br>• Environmental/social planning<br>• Service Provision
	Social Professional Role & Identity	Motivation - Reflective	Education, Persuasion, Incentivization, Coercion	• Communication/Marketing<br>• Guidelines<br>• Fiscal<br>• Regulation<br>• Legislation<br>• Service Provision

Note: As the BCW was mapped to TDF Version 2, the 'Nature of Behaviour' domain is not included. ***Abbreviations:*** Behaviour Change Wheel (BCW); Capability, Opportunity, Motivation – Behaviour (COM-B); Theoretical Domains Framework (TDF).

Table 2) Overall Relevant Intervention Functions & Policies

Interventions		Policies	
• Environmental Restructuring	• Incentivization	• Guidelines	• Service Provision
• Modelling	• Coercion	• Fiscal Measures	• Communication/ Marketing
• Training	• Enablement	• Regulation	• Environmental/Social Planning
• Persuasion	• Education	• Legislation	
• Restriction			

References

1. Michie S, van Stralen MM, West R. The behaviour change wheel: a new method for characterising and designing behaviour change interventions. Implement Sci. 2011;6:42. Epub 2011/04/26. doi: 10.1186/1748-5908-6-42. PubMed PMID: 21513547; PubMed Central PMCID: PMCPMC3096582.
2. Atkins L, Francis J, Islam R, O'Connor D, Patey A, Ivers N, et al. A guide to using the Theoretical Domains Framework of behaviour change to investigate implementation problems. Implement Sci. 2017;12(1):77. PubMed PMID: 28637486; PubMed Central PMCID: PMCPMC5480145.
