# Supplementary material for: Identifying barriers and enablers to rigorous conduct and reporting of preclinical laboratory studies
Source: PLoS Biol. 2023 Jan 5;21(1):e3001932. doi: 10.1371/journal.pbio.3001932 (PMC9888705; doi:10.1371/journal.pbio.3001932)
Supplement: S8 File — (PDF) [file pbio.3001932.s008.pdf]

S8_File: Behaviour Change Wheel and Expert Recommendations for Implementing Change (ERIC) Mapping (Final Consensus)

No.	ERIC Implementation Strategy	BCW Categories	Relevance/Feasibility for Preclinical Researchers
1	Access new funding	Enablement	Included
4	Assess for readiness and identify barriers and facilitators	Enablement	Included
6	Build a coalition	Enablement	Included
7	Capture and share local knowledge	Education, Persuasion, Modelling	Included
8	Centralize technical assistance	Environmental restructuring; Service Provision; Enablement	Included
11	Change physical structure and equipment	Environmental restructuring	Included
14	Conduct cyclical small tests of change	Enablement, Education	Included
15	Conduct educational meetings	Education	Included
16	Conduct educational outreach visits	Education, Persuasion	Included
18	Conduct local needs assessment	Enablement	Included
19	Conduct ongoing training	Training	Included
20	Create a learning collaborative	Enablement, Education	Included
21	Create new clinical teams	Environmental restructuring	Included
23	Develop a formal implementation blueprint	Guideline	Included
24	Develop academic partnerships	Enablement, Training	Included
25	Develop an implementation glossary	Education/ Communication/Marketing	Included
26	Develop and implement tools for quality monitoring	Environmental re-structuring	Included
27	Develop and organize quality monitoring systems	Environmental re-structuring	Included
30	Develop resource sharing agreements	Enablement	Included

31	Distribute educational materials	Education/ Communication/Marketing	Included
33	Facilitation	Enablement (Service Provision for other stakeholders)	Included
35	Identify and prepare champions	Modelling, Training, Education	Included
36	Identify early adopters	Education	Included
38	Inform local opinion leaders	Education	Included
39	Intervene with patients/consumers to enhance uptake and adherence	Enablement	Included
40	Involve executive boards	Enablement	Included
41	Involve patients/consumers and family members	Enablement	Included
43	Make training dynamic	Training, Enablement	Included
44	Mandate change	Persuasion, Guidelines, Communication/Marketing	Included
46	Obtain and use patients/ consumers and family feedback	Enablement	Included
48	Organize clinician implementation team meetings	Enablement, Service Provision	Included
50	Prepare patients/consumers to be active participants	Education, Training, Enablement	Included
51	Promote adaptability	Enablement	Included
52	Promote network weaving	Enablement	Included
53	Provide clinical supervision	Training (*Service Provision for other stakeholders)	Included
54	Provide local technical assistance	Training (*Service Provision for other stakeholders); enablement and environmental restructuring	Included

55	Provide ongoing consultation	Training (*Service Provision for other stakeholders); enablement and environmental restructuring	Included
56	Purposely reexamine the implementation	Enablement	Included
57	Recruit, designate, and train for leadership	Environmental/ Social Planning; Training	Included
59	Revise professional roles	Environmental restructuring	Included
60	Shadow other experts	Modelling	Included
61	Stage implementation scale up	Enablement	Included
63	Tailor strategies	Enablement	Included
64	Use advisory boards and workgroups	Enablement	Included
65	Use an implementation advisor	Enablement	Included
71	Use train-the-trainer strategies	Training	Included
72	Visit other sites	Modelling	Included
73	Work with educational institutions	Enablement, Persuasion	Included
29	Develop educational materials	Education/ Communication/Marketing	Included
69	Use mass media	Persuasion; Communication/ marketing	Included
47	Obtain formal commitments	Regulation, Restriction	Other Stakeholders (Funding Agency, Journals, Educational Institutions, Animal Ethics)
2	Alter incentive/allowance structures	Incentivization, Fiscal Measures, Regulation	Other Stakeholder (Journals, Funding Agency)
5	Audit and provide feedback	Persuasion, Service Provision	Other Stakeholders (Journals, Funding Agency, Implementation Researchers)
9	Change accreditation or membership requirements	Regulation, Restriction	Other Stakeholder (Educational and Animal Ethics Institutions)

10	Change liability laws	Legislation, Coercion	Other Stakeholder (Educational and Animal Ethics Institutions)
12	Change record systems	Environmental restructuring; Environmental /social planning	Other Stakeholder (Journals, Funding Agencies, Animal Ethics Institutions)
22	Create or change credentialing and/or licensure standards	Regulation, Restriction	Other stakeholders (Educational and Animal Ethics Institutions)
28	Develop disincentives	Coercion/ Fiscal measures	Other Stakeholder (Funding Agency)
34	Fund and contract for the clinical innovation	Incentivization/ Fiscal Measures	Other Stakeholders (Funding Agencies)
37	Increase demand	Persuasion	Other Stakeholders (Journals, Funding Agencies)
42	Make billing easier	Environmental restructuring, Fiscal Measures	Other Stakeholder (Funding Agency)
45	Model and simulate change	Education, Persuasion	Other Stakeholder (Implementation Researchers, Educational Institutions)
62	Start a dissemination organization	Enablement, Environmental Restructuring	Other Stakeholder (Journal, Funding Agency, Educational Institution)
66	Use capitated payments	Incentivization/ Fiscal measures	Other Stakeholders (Animal Ethics Institutions)
67	Use data experts	Enablement, Environmental Restructuring, Environmental/social planning	Other Stakeholder (Funding Agency, Journal, Educational Institution, Animal Ethics)

68	Use data warehousing techniques	Environmental restructuring; Environmental/ social planning	Other Stakeholder (Funding Agency, Journal, Educational Institution, Animal Ethics)
58	Remind clinicians	Environmental restructuring; Enablement	Not included because not a relevant TDF domain
3	Alter patient/consumer fees	Fiscal Measures, Incentivization, Coercion	Not applicable for preclinical research
13	Change service sites	Environmental restructuring, Enablement	Not applicable for preclinical research
17	Conduct local consensus discussions	Enablement	Already completed by NIH
32	Facilitate relay of clinical data to providers	Persuasion; Communication/ marketing	Not applicable to preclinical research (and would be burdensome to collect data)
49	Place innovation on fee for service lists/formularies	Incentivization/ Fiscal measures	Not applicable for preclinical research
70	Use other payment schemes	Incentivization/ Fiscal measures	Not applicable for preclinical research
