# Supplementary material for: Identifying barriers and enablers to rigorous conduct and reporting of preclinical laboratory studies
Source: PLoS Biol. 2023 Jan 5;21(1):e3001932. doi: 10.1371/journal.pbio.3001932 (PMC9888705; doi:10.1371/journal.pbio.3001932)
Supplement: S9 File — (PDF) [file pbio.3001932.s009.pdf]

S9_File: Organization and Categorization of Expert Reccomendations for Implementing Change (ERIC) Implementation Strategies

Implementation Phases	Education / Persuasion Phase	
**Preparation**	**Scoping current practices**<br>**Assess barriers / enablers**	Identify early adopters<br>Capture/share local knowledge<br>Conduct local needs assessment<br>Assess for readiness and identify barrier and facilitators<br>Shadow other experts<br>Visit other sites
**Capacity Building**	**Partnering for change**<br>**Developing materials**	Work with educational institutions<br>Inform local opinion leaders<br>Develop an implementation glossary<br>Develop educational materials<br>Use Mass Media<br>Access new funding
**Implementation**	**Educational outreach**<br>**Distribute materials**	Conduct educational outreach visits<br>Conduct educational meetings<br>Create a learning collaborative<br>Distribute educational materials

------>

Implementation Phases	Training / Modelling Phase	
**Preparation**	**Involve stakeholders**<br>**Develop formal plan**	Develop academic partnerships<br>Organize clinician implementation team meetings<br>Develop a formal implementation blueprint<br>Obtain and use patients/consumers and family feedback<br>Involve patients/consumers and family members<br>Use an implementation advisor
**Capacity Building**	**Identify and prepare local champions**	Identify and prepare champions<br>Use train-the-trainer strategies<br>Prepare patients/consumers to be active participants
**Implementation**	**Cyclical tests of change**<br>**Ongoing feedback**	Make training dynamic<br>Conduct ongoing training<br>Provide ongoing consultation<br>Provide clinical supervision<br>Provide local technical assistance<br>Conduct cyclical small tests of change<br>Intervene with patients/consumers to enhance uptake and adherence

------>

Implementation Phases	Environmental Restructuring Phase	
**Preparation**	**Institutional involvement**<br>**Tailored strategies**	Involve executive boards<br>Build a coalition<br>Use advisory boards and workgroups<br>Mandate change<br>Tailor strategies
**Capacity Building**	**System development**<br>**Building teams**	Develop / implement tools for quality monitoring<br>Develop and organize quality monitoring systems<br>Develop resource sharing agreements<br>Create new clinical teams<br>Revise professional roles<br>Centralize technical assistance<br>Recruit, designate, and train for leadership
**Implementation**	**Staged scaling**<br>**Promote adaptability**	Change physical structure and equipment<br>Stage implementation scale up<br>Promote network weaving<br>Promote adaptability<br>Facilitation<br>Purposefully reexamine the implementation
