# Supplementary material for: Identifying barriers and enablers to rigorous conduct and reporting of preclinical laboratory studies
Source: PLoS Biol. 2023 Jan 5;21(1):e3001932. doi: 10.1371/journal.pbio.3001932 (PMC9888705; doi:10.1371/journal.pbio.3001932)
Supplement: Attachment — Submitted filename: Response to Reviewers (Nov 2022)_FINAL.docx [file pbio.3001932.s011.docx]

**REVIEWERS' COMMENTS:

Reviewer #1:**

**Summary of research**

**"Preclinical guidelines" such as ARRIVE inform researchers conducting (animal) experiments and readers of (their) scholarly publications on matters of scientific rigour. They further enable reviewers and readers to adequately assess and evaluate the methodological robustness of the research study presented to ensure its reproducibility.
Referring to the poor adoption of such guidelines by preclinical researchers, the study presented by Manoj et al. aims to
A) identify factors influencing the application of "preclinical guidelines" and
B) derive implementation strategies, measures and policies for research-funding organizations and/or research-performing organizations to improve reproducibility in preclinical research.

Hence, the manuscript's subject matter is relevant to the research community and target groups PLOS Biology addresses.

Manoj et al. chose a qualitative research design interviewing thirty expert researchers working in cancer and cardiovascular research. To identify factors that influence the adoption of research practices (standards, replication, statistics, randomization, blinding, sample estimation, inclusion and exclusion criteria derived from NIH principles and guidelines for reporting preclinical research) supposed to improve the reproducibility of preclinical research, the authors addressed 14 behavioural domains synthesized from 128 constructs derived from 33 theories of behaviour and behaviour change clustered the 'Theoretical Domain Framework', TDF, [Ref. 43] using a semi-structured interview guide.

Manoj et al. used a deductive approach and coded interviewees' quotes based on TDF domains but determined their relevance through quantitative, here frequency analysis. Quotes were further aggregated in overarching "belief statements" representing their meaning and clustered in one "enabler theme" and two "barrier themes". (A)

To identify appropriate implementation strategies, they mapped TDF domains identified as relevant to the "Behavioral Change Weel" [Ref 21, 29] and the "Expert Recommendation for Implementing Change" project [Ref 28],  with the latter referred to as two tools for selecting/designing implementation strategies that lead to behavioural change in individuals. (B)

Briefly, behavioural change requires that individuals become aware of a problem that gives them a 'reason' to reconsider a particular behaviour. The individual may weigh the costs and benefits of alternative behaviours and form an attitude (consisting of cognitive, affective, and normative evaluative elements) that leads to the intention to make a decision, either to change or to maintain a particular behaviour. Importantly, behavioural change usually occurs in a social setting (team, department, organization) and is supposed to be followed by various social and contextual responses that may or may not reinforce the behaviour. Last but not least, external factors such as organizational/national/supra-national policies or funding mechanisms influence the interviewees' current research practices addressed in the study.**

**Major issues with regard to A)

To get deeper insights and understand the interviewees' current behaviour /behavioural intentions intention, the authors may consider presenting case summaries based on a code-document/case-matrix and performing/presenting a qualitative case comparison analysis. Further, Manoj et al. could apply a more data-driven approach, i.e. inductive development of categories and codes.**

RESPONSE: We thank the reviewer for their careful consideration of our work. While we agree that alternative analytical methods could be taken to interrogate these qualitative data, we were deliberate in selecting the Theoretical Domains Framework to inform both our data collection and our analysis to enable us to draw upon a well-established set of methodological approaches for identifying types of barriers/enablers for specific behaviours and then linking these to fit-for-purpose strategies [1, 2]. We feel that this approach to analysis, enabling a connection to the wider methodological literature and connection to particular strategies for addressing barriers and enablers, provides both a novel approach to improving preclinical researchers’ study design and reporting practices, while also providing a rigorous foundation to inform future interventions. Our approach draws from established methodological literature and applies it to a novel setting, provides a lens into interviewees’ current behaviours/intentions and a clear path forward to inform how to support them in changing their practice in the future.

We also note that while our study was designed and conducted using a primarily deductive approach, the latter portion of our analysis (development of global sub-themes and themes) were in effect inductively generated; thus, there remains an inductive element to global theme and sub-theme generation.

1. Atkins L, Francis J, Islam R, O'Connor D, Patey A, Ivers N, et al. A guide to using the Theoretical Domains Framework of behaviour change to investigate implementation problems. Implement Sci. 2017;12(1):77. PubMed PMID: 28637486.

2. Michie S, van Stralen MM, West R. The behaviour change wheel: a new method for characterising and designing behaviour change interventions. Implement Sci. 2011;6:42. Epub 2011/04/26. doi: 10.1186/1748-5908-6-42. PubMed PMID: 21513547.

**Major issues with regard to B)

Considering the study's limited sample size, Manoj et al. would need to provide information on how many research-performing organizations the interviewees represent and whether preferentially Canadian or international organizations were chosen. With respect to the complexity of (social) behaviour, the authors would need to provide a plausible argumentation of their generalization claims concerning the identification of organizational implementation strategies.**

RESPONSE: The interviewees were primarily affiliated with our funders (two federally funded Canadian research networks), thus our final sample included 29 Canadian researchers and one American researcher from 13 different institutions. For a qualitative study, we rather feel that our sample size of n=30 would be seen as a strength in terms of size and scope and diversity of perspectives spanning gender, career level, main areas of research and years of experience.

We appreciate the point raised about generalisability but our approach to qualitative research draws not on the more statistical-probabilistic concept of generalisability that underpins most quantitative research. Rather, we draw on how qualitative research typically approaches the concept of generalisability as described by Smith in his highly cited exposition on this topic [1].

The first relates to *transferability,* i.e. whether results are transferable to other settings. Given that Canadian biomedicine follows an archetype of academic research that is similar to the US and much of Europe (i.e. in terms of funding, lab organization, institutional support, and incentives that drive academic researchers), we believe our paper establishes a foundation for a variety of labs to draw upon to further develop implementation strategies best suited to their settings.

In terms of the organisational implementation strategies suggested in our study – this speaks to *generativity* in that we “invite [readers] into an experience and move them to act upon what they read…” [1]. Based on the transferability of our findings, we believe our strategies identify feasible steps towards intervention development from which readers in a position to act on them can consider drawing from. As suggested in our manuscript, development of larger scale implementation strategies will require an even broader set of stakeholders to further refine the list of strategies and identify which are best suited to which organisational contexts (cf tailoring). While that latter development is beyond the scope of this specific paper/study, we hope our paper establishes a foundation for us and others to draw upon to further develop implementation strategies best suited to different settings. This approach for future work in this area would be consistent with the iterative nature of any implementation intervention development [2, 3].

Finally, based on dissemination of our findings to date through scientific meetings and to ‘knowledge user’ organizations, we believe our work also demonstrates *naturalistic generalizability,* where our results resonate with the audience’s personal experiences [1]. Indeed, when discussing this work with a variety of basic scientists and institutional leaders, we have frequently received feedback that the results and themes we’ve identified ‘ring true’ with them. Moreover, following a discussion with NIH policy analysts, we were asked to share our results with the NIH Office of Science Policy; again, we believe this is a demonstration of naturalistic generalization.

We have updated the Strengths and Limitations section to the following:

*“We also acknowledge that a limitation of our study is a focus largely on Canadian academic institutions (reflected by sampling through our two Canadian funders). Nonetheless, Canadian biomedicine does follow an archetype of academic research that is widely adopted (i.e. in terms of funding, lab organization, institutional support, and incentives that drive academic research). Further research evaluating the feasibility of implementing such behavior change strategies, across different institutions, as well as the potential role of other key stakeholders, is warranted.”*

1. Smith B. Generalizability in qualitative research: misunderstandings, opportunities and recommendations for the sport and exercise sciences, *Qualitative Research in Sport, Exercise and Health* 2018; 10:1,137-149, doi: [10.1080/2159676X.2017.1393221](https://doi.org/10.1080/2159676X.2017.1393221)
2. Craig P, Dieppe P, Macintyre S, Michie S, NazarethI, Petticrew M et al. Developing and evaluating complex interventions: the new Medical Research Council guidance  *BMJ*  2008;  337 :a1655 doi:10.1136/bmj.a1655
3. Skivington K, Matthews L, Simpson S A, Craig P, Baird J, Blazeby J M et al. A new framework for developing and evaluating complex interventions: update of Medical Research Council guidance  *BMJ* 2021;  374 :n2061 doi:10.1136/bmj.n2061

**Other major issues

To strengthen the transparency of the study and ensure the reproducibility of the authors' analysis, it would be helpful for readers and reasonable for researchers who may want to re-analyze or re-use the study's data if Manoj et al. share their data. Among other things, this includes anonymized interview transcripts (approved by interviewees), the study's final category system (coding frame), a code-document matrix, and a detailed analysis protocol.**

RESPONSE: We believe we have followed as many principles of open data as is feasible. We would also note that questions around open data in qualitative research remain far more contested than in quantitative research. There are a number of epistemological, methodological, and ethical/legal issues that remain to be resolved (see Chauvette et al for an exposition of these significant issues [1]).

We are not able to share the anonymized interview transcripts as this was not specified in our Research Ethics Board approval or consent forms. We agree the data availability statement should be updated to reflect this. It has now been updated to: *“Data are available in the main text or the supplementary materials. Data and materials pertaining to individual participants will not be shared to protect privacy.”*

In regard to the study’s final category system (coding frame), the team outlined the behaviour of interest using the AACTT framework [1] (Supplemental File #3) and coded according to established, published guidance outlined by Michie et al 2005, Cane et al 2012 and Atkins et al 2017. Interview transcripts were coded using the pre-existing domains from Theoretical Domains Framework (TDF) Version 2, in addition to one domain from TDF Version 1 (Nature of Behaviour). Definitions provided by the associated papers and guide were used (brief descriptions are outlined in Supplemental File #1). Within the Methods section of the paper we have now provided these further details to clarify our approach.

We have now updated the ‘Data Analysis’ section to the following:

*“Transcripts were imported to the qualitative software program NVivo 11. We performed directed content analysis [21], wherein the TDF domains were used to organize and codify the data. First, quotes from the interview transcripts were assigned to the relevant TDF domains [43]. Quotes could be assigned to multiple domains if it was felt that more than one domain was represented. The first 11 interviews were codified in duplicate by two independent researchers (MF + AC or VH), and any conflicts were resolved through a consensus discussion. The remaining 19 interviews were then codified by one researcher (MF). All coders were trained in application of the TDF to interview studies. In the second stage of analysis, the codified data was reviewed according to each TDF domain. Within each domain, similar quotes were grouped together and described using an overarching ‘belief statement’ (specific belief representing the underlying meaning of the quotes). Similar belief statements were also grouped together to create broader themes. In step three, the results were reviewed to determine which TDF domains were most relevant to the behavior of interest (i.e., to adopt preclinical guidelines). This analysis was based on frequency of beliefs, strength of beliefs and conflicting beliefs as outlined by Patey et al [44]. Once relevant domains were identified, global themes were generated to describe high level, interesting features of the data, spanning across the domains. Tables were created to display the identified global themes, sub-themes, belief statements, frequency of these belief statements and the relevant domains (Supplemental File 5). An overview of our study design and analysis approach can be found in Figure 2.”*

In our Supplemental Files we have included:

- Definition of the behaviour of interest (e.g. action, actor, context, target and time) (Supplemental File #3)
- The interview guide and handout (Supplemental File #4)
- Description of Theoretical Domains Framework Domains used to codify the data (Supplemental File #1)
- Subthemes for each of the 4 global themes, example belief statements/frequencies, and the associated TDF domains (Supplemental File #5).
- More detailed description of the mapping exercise methods and results (Supplemental File #6)
- Mapping of the TDF domains to the Behaviour Change Wheel intervention functions and policies (Supplemental File #7)
- Overall relevant intervention functions and policies (Supplemental File #7)
- Our coding of the ERIC Implementation strategies to the Behaviour Change Wheel, and relevance/feasibility for preclinical researchers (Supplemental File #8)
- Organization and categorization of the ERIC implementation strategies by implementation stage (#Supplemental File 9)

The team posted a study protocol on Open Science Framework (OSF; https://osf.io/dth7b/). More detailed methods can be found in the Methods section of the manuscript. As mentioned, we have made edits to clarify our approach (as guided by Michie et al 2005, Cane et al 2012 and Atkins et al 2017).

1. Chauvette, A., Schick-Makaroff, K., & Molzahn, A. E. Open Data in Qualitative Research. *International Journal of Qualitative Methods* 2019; 18, <https://doi.org/10.1177/1609406918823863>
2. J. Presseau, N. McCleary, F. Lorencatto, A. M. Patey, J. M. Grimshaw, J. J. Francis, Action, actor, context, target, time (AACTT): a framework for specifying behaviour. *Implement Sci* 2019; **14**, 102.

**Reviewer #2:**
**There is a real need to explore how to drive change to implement robust experimental design as standard in the in vivo space.  This manuscript looks to explore the blockers through qualitative research and provide a framework to move the community forward. As a scientist working actively in this area,  this manuscript should have high interest to me.  However,  it didn't quite land and the analysis of the interviews seemed superficial and uninformative.  The paper was also not cohesive  and this could be domain specialist not articulating clearly enough to researcher from other fields.  There are also some sections that could perpetuation misunderstandings and hinders the implementation of robust experimental design.**

**Major:**
**1.  This article will be read by scientist who are not qualitative research specialist.  It needs to be assessable to this audience.  Review the text to ensure accessibility for the target audience. Please also include a schematic of the workflow to give a big picture explanation of the work presented in this article e.g. Survey construct focused on TDF domains - survey - coding  - summation of observations - Behavioural wheel.**

RESPONSE**:** We thank the reviewer for recognizing the need for studies in this area. We agree that an explanatory approach for non-qualitative researchers is needed. We have now reviewed the manuscript and made edits throughout to improve readability. We have also now:

- Included a schematic of the workflow to provide an overview and explanation of the work presented in the article (Figure 2).

- Moved more detailed results from mapping of the interview findings to the Behaviour Change Wheel to Supplemental File 6 (and adjusted the numbering of subsequent files accordingly)

**2.  The paper for a non expert isn't cohesive.  For example
A:  In supplementary file 1:  there are two separate tables both describing domains but in different ways.  One has a series of definitions and  the other is a series of questions.  I don't understand how these were used in the analysis.  I original though that the first set were used to classify the response provided to the survey question…**

RESPONSE: Thank you for this suggestion to improve understanding. We have made an adjustment to Supplementary File #1 to collate all information into one simplified table. We have also clarified that this table encompasses definitions of the domains used to codify the data.

**…. I then don't understand how nature of the behaviour fits into this process.**

RESPONSE: Apologies for the confusion. In total 15 domains were considered. We have clarified this in Supplemental File 1 in an explanatory manner as suggested by the reviewer.

*“As a first step to organizing and analyzing our interview study data, using a deductive approach, we applied the Theoretical Domains Framework (TDF) to codify the data.^1^ We considered 15 domains of the TDF (i.e. all 14 domains from TDF version 2 as well as the ‘nature of the behavior’ from TDF version 1). We have provided definitions for each domain below.”*

In the Table we have updated the description of ‘Nature of Behavior’ to: *“Dimensions on which desired behaviours might vary”*

**B: Supplement File 4:   We now have construct statement associated with each section.  Sometimes this overlaps with the TDF domains but sometimes doesn't.  How does this fit in?**

RESPONSE: Supplementary File 4 encompasses the interview guide questions. The headers are meant to demonstrate which domain the questions are intended to address. Related theoretical constructs associated with each domain are found in brackets in the header; these are meant to aid the research assistants when conducting and analyzing the interviews (as per Michie et al 2005, Cane et al 2012 and Atkins et al 2017). We have now provided an explanatory paragraph at the top of the supplemental file to ensure that this is clear.

*“****Note:*** *Headers throughout the main interview questions are meant to demonstrate which domain the questions are intended to address, along with related theoretical constructs associated with each domain. These were meant to aid research assistants when conducting and analyzing the interviews.”*

**3.  Explorative versus confirmation experiments.**
**This is a hazardous area.  There is not consensus on the definitions.   For some communities,  if you class the work as explorative then you should not apply any statistical tests.  In my experience, majority of research is explorative and not confirmation (where you are looking to replicate a known ES).  The current wording states that if you are in explorative mode you cannot do a power calculation.  That is not true.    Consider an omic experiment using ex-vivo samples,  we still conduct power analysis we input a SD metric that encompasses 75% of the variables of interest and define an effect size of interest.  Alternatively,  where you have multiple outcome variables,  you can talk about power in terms of cohen's d and reflect on predicted ES for this intervention.  The paper is contributing to the misunderstanding around power calculations.**

RESPONSE: We agree with the reviewer that this is a problematic area. Indeed, defining and differentiating exploratory studies from confirmatory studies is an active area of research in our group. We have **deleted** the sentence that was meant to describe sentiments from the interview participants: *~~“For instance, in exploratory research the primary outcome may not be defined and effect sizes are unknown; thereby, making it impossible to use a power calculation to compute the sample sizes.”~~*

In addition, we have now added the below sentence on page 6 to clarify.

*“These themes are presented alongside illustrative quotes that demonstrate participant beliefs and do not represent the position of the authors on each subject.”*

**3. Inclusion and exclusion criteria.**
**The definition includes both the criteria around animals and data.  On page 8,  a quote  is included that says it isn't relevant because they are in bred mice.  This is focused only on one aspect of the criteria - the animal not the data.  The presentation in this section is at real risk of perpetuating the misunderstanding of the definition and intention around this criteria.**

RESPONSE: We agree with the reviewer that this may lead to misunderstanding. We have made several changes to address this issue.

We have **deleted** the original quote as well as the sentence that was describing the original quote:

*~~“so we don’t apply [inclusion and exclusion criteria] very often… because the models that we use are inbred. So they’re all identical. So I’m not really sure how you would have inclusion and exclusion criterias compared to like humans, we’re all different. So we have to make sure that the population of people that we’re studying are as homogeneous as possible. Where mice are already, by definition, homogeneous.”~~* ~~– Participant 8~~

*~~“One participant expressed that inclusion/exclusion criteria is not as relevant in preclinical in vivo research, as the mice used are in-bred. Despite this…”~~*

The word ‘still’ was also removed from the subsequent sentence so that it reads: *“A priori inclusion and exclusion criteria are important for in vivo experiments; epigenetic and environmental factors can lead to differences in animal populations.”*

We have also now added the below quote to encompass the data aspect of inclusion/exclusion criteria:

*“…we do have inclusion and exclusion criteria, but they tend to be, post analysis, not, we don’t normally decide them, before the start of the experiment because all of the stuff we do is kind of new and sometimes we don’t really know what to expect, right, we cross the bridge when we get there. So it’s, I would say inclusion/exclusion criteria might be, might be something that we could improve...”-* Participant 18

We have added the below sentence on page 6 to clarify:

*“These themes are presented alongside illustrative quotes that demonstrate participant beliefs and do not represent the position of the authors on each subject.”*

**4:  Superficial reflection**
**Examples**
**a: The survey seems very interest and explores many different drivers of behaviour e.g. self efficacy etc but this doesn't then come through in the evaluation.  If the survey results were framed in the language of construct or TDF - this would add more reflection on the drivers behind this behaviour.  Just is saying what we already know.  Adding more reflection on the drivers would have been interesting.**

RESPONSE: Throughout generation of belief statements, sub-themes and global themes, we aimed to keep the language accessible to a wide audience. To further reflect on drivers, we have now added the below sentence to the discussion:

*“Two recent papers assessing perspectives, barriers and enablers to rigorous reporting in preclinical research (one focused on complete reporting, while the other looked at preregistration of animal studies) came to similar conclusions; various stakeholders involved in preclinical research may be able to encourage implementation of these behaviours by collaborating and creating a system that rewards or incentivizes (e.g. influences hiring, mandates) [41,42]. We believe our finding that researchers are motivated to implement the guidelines, yet continue to face resource and education related challenges in doing so, also suggests action by additional stakeholders will be required.”*

**b: Randomisation.  During the interview the researchers said they were confident to roll out these methods and very often had experience of doing it.  However, the paper highlights that the randomisation implemented wasn't true randomisation.  The missing point - is that the scientists are over stating their abilities - probably due to a lack of knowledge. This means the self reporting is also a weakness of this analysis.**

RESPONSE: We agree that knowledge gaps are a significant issue and we highlight this in our Discussion. The reviewer actually points out a significant advantage of this qualitative, interview approach – through probing and discussion with the interviewees we were able to identify these knowledge gaps. However, we agree that self-reporting could present limitations. We have now added the below sentence to the Strengths and Limitations section:

*“First, as with any interview-based study, our results depend on self-reporting. We acknowledge that our results may be influenced by social desirability bias, wherein responses may lean towards what participants feel the interviewer would like to hear. We aimed to address this by emphasizing at the beginning of the interview that there are no ‘right’ or ‘wrong’ answers, and that we were simply interested in interviewee perspectives to help inform the design of resources.”*

**c.      The scientists stated they were confident and yet most do not routinely make the distinction between biological and technical replicates.  This process doesn't have direct practical costs but significant statistical implications.  If they are confident they should do it,  and they believe in the value then they should do it.  If they are not, then it probably due to lack of analysis skills etc.**

RESPONSE: We agree with the reviewer. We have now made an addition/adjustment in the discussion to include the following:

*“We also note that implementation of certain aspects of the guidelines do not take any resources, but have significant implications on the statistics and analysis of experiments, such as the distinction between biological and technical replicates. We therefore similarly suggest that educational initiatives underscoring this information would be helpful.”*

**d.       We know a lot of these issues - none of this is a surprise.  To drive change we need hard number.    E.g. "multiple participants shared they had not received formal training" what proportion?   What proportion had not heard of any guidelines?**

RESPONSE: We note that as a part of our analysis, we did include frequency counts for belief statements (Supplementary File #5). However, we have not reported these numbers in the text to avoid suggesting that these are representative proportions. Though frequency counts can be captured- their use in qualitative research remains contentious, as the core goal of this type of research is to better understand participant perspectives within their social contexts, not provide definitive numbers on the prevalence of different responses as one would with quantitative research. Nonetheless, we do appreciate that readers may be interested in frequency of statements and we have now made it clearer that these can be found in the supplemental files:

*“Four global themes were identified, two that reflect barriers and two that reflect enablers (themes, sub-themes, relevant TDF domains, specific beliefs and associated frequency counts are presented in Supplemental File 5).”*

**e.      "Most stated they were very confident or confident in their capabilities to apply all the experimental design methodology" - OK if that was true then it is at odds with a lot of other details that are presented.  For example asking for training,  asking for statisticians etc.  This paper has no reflection on these conflicting observations. Is this arising from peoples first reaction to reflecting on change is to throw out reasons why it can't be done and it impossible and the process of you asking is leading to them feeling actually they could do it and really should.  The quotes suggests your interview took people on that journey.  How does that help us think about the next steps?**

RESPONSE: We agree that this is a jarring contrast between perceived abilities and application of these methods. We would suggest that this may be due to knowledge gaps (i.e. the researchers may overestimate their abilities, as the reviewer has also pointed out) as well as resource constraints.

We have now highlighted this further in the Discussion (page 16).

*“Conflicting observations across our study findings (e.g. high motivation and confidence in implementing the guidelines, but numerous noted challenges and need for further resources/support) may suggest possible overestimation of abilities and resource constraints.”*

In terms of next steps, we believe these conflicting observations highlight the need for action by additional stakeholders (e.g. funding agencies, journals). We have now included recent examples of funding calls that may help spur practice change (page 18).

**4:  This paper is structured around the NIH guidelines.  Why then does the abstract reference the ARRIVE guidelines?**

RESPONSE: We agree that this sentence in the abstract was confusing – this wording has now been removed so that the sentence reads:

*“Despite promotion of these practices by funders and journals, assessments suggest uptake is low in preclinical research.”*

**5:  Limitations - I assume all these researchers are academic?  US based?  This isn't presented in table 1 and needs discussing in the limitations section.**
RESPONSE**:** We thank the reviewer for noting this. We have now highlighted this in the Results and Limitations.

Results: *“Interviewees were primarily affiliated with our funders (two federally funded Canadian research networks), thus our final sample included 29 Canadian researchers and one American researcher from 13 different institutions.”*

Limitations: *“We also acknowledge that a limitation of our study is a focus largely on Canadian academic institutions (reflected by sampling through our two Canadian funders). Nonetheless, Canadian biomedicine does follow an archetype of academic research that is widely adopted (i.e. in terms of funding, lab organization, institutional support, and incentives that drive academic research). Further research evaluating the feasibility of implementing such behavior change strategies, across different institutions, as well as the potential role of other key stakeholders, is warranted.”*

**Minor
1.      Supplementary File 1:  behavioural regulation - the definition was not accessible to me.**

RESPONSE**:** To avoid altering the American Psychological Association Dictionary definition (as originally reported in *‘Validation of the theoretical domains framework for use in behaviour change and implementation research ’* by Cane et al. (2021)), we have aimed to provide a more accessible definition by removing unnecessary words. We have now updated the definition to:

*“Anything aimed at managing or changing […] actions”*

**2.      When talking about guidelines what about PREPARE?**

RESPONSE: We have now added the PREPARE guidelines to the sentence on page 18. The sentence now reads:

*“From this evidence, it is conceivable that planning and reporting of preclinical experiments could be improved if journals were to adopt and enforce harmonized reporting guidelines such as the NIH guidelines [12], ARRIVE 2.0 guidelines [1, 30], or PREPARE [44].”*

**3.      ARRIVE 2.0 has a essential set for journals to prioritise - this should mentioned in the section where this is discussed.**

RESPONSE: We have now updated the sentence on page 3 to the following: *“Though labelled as a reporting guideline, as emphasized by the similar Animal Research: Reporting of In Vivo Experiments (ARRIVE) guideline and the recent 2020 ARRIVE 2.0 guideline…”*

As above we have added the ARRIVE 2.0 guidelines to the sentence on page 18. The sentence now reads:

*“From this evidence, it is conceivable that reporting of preclinical experiments could be improved if journals were to adopt and enforce harmonized reporting guidelines such as the NIH guidelines [12], ARRIVE 2.0 guidelines [1, 30], or PREPARE [44]. ARRIVE 2.0, in particular has a set of “essential 10” elements that could be emphasized by stakeholders. ”*

**4.      Table 2  - "Look for and apply to funding opportunities prioritizing practices...." I found it hard to parse and wasn't sure what you meant.  Do you mean apply to funding bodies that require you to follow good practice as default?**

RESPONSE: We had meant to suggest that researchers could focus on applying to funding opportunities that explicitly value and reward good practice. We have updated this sentence to the below to clarify:

*“Identify funding opportunities that require researchers to prioritize practices outlined by design and reporting guidelines”*

**5.      Quite a few sentences are hard to parse eg**
**a.      "As we only conducted interviews with preclinical researchers … " page 15.**

RESPONSE**:** This sentence has been reworded to: *“These implementation strategies focus on providing researchers with a wide array of specific actions that they can implement (e.g. at the investigator and/or trainee level).”*

**b.      "Nonetheless, the feasibility issues associated with the practices discussed by …" page 16.**

RESPONSE**:** The sentence has been reworded so that it reads: *“Nonetheless, the feasibility issues associated with the practices are valid concerns as laboratories are already constrained by time and resources.”*

**6.      Data availability - I would dispute that all data has been made available as the transcripts have not.  Please reword.**

RESPONSE**:** We agree. This issue around data availability is qualitative research is a contentious one [1]. We have now reworded to: *“Data are available in the main text or the supplementary materials. Data and materials pertaining to individual participants will not be shared to protect privacy.”*

1. Prosser AMB, Hamshaw RJT, Meyer J, et al. When open data closes the door: A critical examination of the past, present and the potential future for open data guidelines in journals [published online ahead of print, 2022 Sep 8]. Br J Soc Psychol. 2022;10.1111/bjso.12576. doi:10.1111/bjso.12576

**Reviewer #3:
[identifies himself as Timothy M. Errington]

The manuscript by Lalu et al. describes results from multiple structured interviews from preclinical researchers (specifically cancer and cardiovascular researchers who perform in vivo research) on the topic of implementing and reporting preclinical research guidelines. They identified four global theme from these interviews (two barriers and two enablers) that identify opportunities to inform strategies and approaches in improving the conduct and reporting of preclinical research.

Overall, I thought this was a well written manuscript on an important and timely topic that will be of interest to a broad audience. I enjoyed reading it and the excerpts from the interviews give great context to what the authors found in their study. Below are suggests for the authors to consider in improving their manuscript:

[1] The authors recruited from Canadian funders, does that mean the researchers who participated were also from Canadian institutions or were they more geographically distributed? Similarly, how broad were the participants in terms of institutions (e.g., were many at the same institution or were all from different institutions)? The reason is how broadly applicable these points of view are. As the authors indicated a limitation of the current study is that it only included cancer and cardiovascular researchers, but it is likely more limited than that – such as only academic researchers (e.g., were industry researchers invited) at Canadian institutions. Any additional information on this could be included in the methods, limitations section, and table 1 where applicable.**

RESPONSE: We thank Dr. Errington for his positive assessment of our study and manuscript. This first response was also provided to Reviewer #1.

The interviewees were primarily affiliated with our funders (two federally funded Canadian research networks), thus our final sample included 29 Canadian researchers and one American researcher from 13 different institutions. For a qualitative study, we rather feel that our sample size of n=30 would be seen as a strength in terms of size and scope and diversity of perspectives spanning gender, career level, main areas of research and years of experience.

We appreciate the point raised about generalisability but our approach to qualitative research draws not on the more statistical-probabilistic concept of generalisability that underpins most quantitative research. Rather, we draw on how qualitative research typically approaches the concept of generalisability as described by Smith in his highly cited exposition on this topic [1].

The first relates to *transferability,* i.e. whether results are transferable to other settings. Given that Canadian biomedicine follows an archetype of academic research that is similar to the US and much of European (i.e. in terms of funding, lab organization, institutional support, and incentives that drive academic researchers), we believe our paper establishes a foundation for a variety of labs to draw upon to further develop implementation strategies best suited to their settings.

In terms the organisational implementation strategies suggested in our study – this speaks to *generativity* in that we “invite [readers] into an experience and move them to act upon what they read…” [1]. Based on the transferability of our findings, we believe our strategies identify feasible steps towards intervention development from which readers in a position to act on them can consider drawing from. As suggested in our manuscript, development of larger scale implementation strategies will require an even broader set of stakeholders to further refine the list of strategies and identify which are best suited to which organisational contexts (cf tailoring). While that latter development is beyond the scope of this specific paper/study, we hope our paper establishes a foundation for us and others to draw upon to further develop implementation strategies best suited to different settings. This approach for future work in this area would be consistent with the iterative nature of any implementation intervention development [2, 3].

Finally, based on dissemination of our findings to date through scientific meetings and to ‘knowledge user’ organizations, we believe our work also demonstrates *naturalistic generalizability,* where our results resonate with the audience’s personal experiences [1]. Indeed, when discussing this work with a variety of basic scientists and institutional leaders, we have frequently received feedback that the results and themes we’ve identified ‘ring true’ with them. Moreover, following a discussion with NIH policy analysts, we were asked to share our results with the NIH Office of Science Policy; again, we believe this is a demonstration of naturalistic generalization.

We have updated the Strengths and Limitations section to the following:

*“We also acknowledge that a limitation of our study is a focus largely on Canadian academic institutions (reflected by sampling through our two Canadian funders). Nonetheless, Canadian biomedicine does follow an archetype of academic research that is widely adopted (i.e. in terms of funding, lab organization, institutional support, and incentives that drive academic research). Further research evaluating the feasibility of implementing such behavior change strategies, across different institutions, as well as the potential role of other key stakeholders, is warranted.”*

1. Smith B. Generalizability in qualitative research: misunderstandings, opportunities and recommendations for the sport and exercise sciences, *Qualitative Research in Sport, Exercise and Health* 2018; 10:1,137-149, doi: [10.1080/2159676X.2017.1393221](https://doi.org/10.1080/2159676X.2017.1393221)
2. Craig P, Dieppe P, Macintyre S, Michie S, NazarethI, Petticrew M et al. Developing and evaluating complex interventions: the new Medical Research Council guidance  *BMJ*  2008;  337 :a1655 doi:10.1136/bmj.a1655
3. Skivington K, Matthews L, Simpson S A, Craig P, Baird J, Blazeby J M et al. A new framework for developing and evaluating complex interventions: update of Medical Research Council guidance  *BMJ* 2021;  374 :n2061 doi:10.1136/bmj.n2061

**[2] On page 9 a common concern was expressed by participants that the rigor elements in the reporting guidelines (e.g., increased sample sizes) would lead to less experiments, which would be perceived as a decrease in productivity (e.g., less papers). This tension (e.g., many lower quality experiments vs less higher quality experiments) is a central factor not currently discussed in the paper. The discussion includes how funders and journals could adopt strategies to encourage implementation of guidelines and core practices, but not how to shift this incentive concern being raised that is a major barrier to adoption.**

RESPONSE: We agree that skewed incentives (e.g. quantity of papers tied to promotion) almost certainly underly some of the issues identified. We have now highlighted funding calls that prioritize and reward use of these methods to address cost and resource concerns (as outlined on page 16-18):

a) *“Current systems largely reward quantity (e.g. of published papers) over the rigour and reporting. Thus, funders could spearhead more initiatives that highlight and reward implementation of practices to improve rigour and reporting. Ultimately, such rigour and transparency will facilitate proper evaluation by the scientific community. Notably, despite costs, constraints and variable knowledge, participants were motivated to implement the core practices as they believed that they would lead to positive outcomes and benefits. These outcomes were chiefly concerned with ‘good scientific practice’ and increasing the quality of their work and trust in their findings. This further supports the notion that with education, training, support, and incentives researchers would be likely to implement core practices.”*
b) *“As examples of these strategies in action, BMBF, a German funding agency, has recently issued a funding competition specifically for preclinical “confirmatory” studies [45], and NIH has also issued several funding competitions focused on improving education in principles of experimental rigor [46]. Institutions may also be able to build on the Declaration on Research Assessment (DORA) initiative [47] by signing and agreeing to base policies that reward quality (over other quantity-based metrics). ”*

References:

45. Bundesministerium fur Bildung und Forschung [Federal Ministry of Education and Research]. Announcement [June 22, 2022; cited October 23, 2022]. Available from: [*https://www.bmbf.de/bmbf/shareddocs/bekanntmachungen/de/2022/07/2022-07-20-Bekanntmachung-Reviews.html?view=renderNewsletterHtml*](https://www.bmbf.de/bmbf/shareddocs/bekanntmachungen/de/2022/07/2022-07-20-Bekanntmachung-Reviews.html?view=renderNewsletterHtml)

46. National Institute of Neurological Disorders and Stroke. Initiative to Improve Education in the Principles of Rigorous Research [August 4, 2022; cited October 23, 2022]. Available from: [*https://www.ninds.nih.gov/current-research/trans-agency-activities/rigor-transparency/initiative-improve-education-principles-rigorous-research*](https://www.ninds.nih.gov/current-research/trans-agency-activities/rigor-transparency/initiative-improve-education-principles-rigorous-research)*)*

47. Declaration on Research Assessment (DORA). About DORA [Cited October 23, 2022]. Available from: *https://sfdora.org/about-dora/*

**Minor points:

[1] In the abstract, listing the themes (e.g., Barrier Theme 1) seems unnecessary as the context is missing. I think the authors could delete these as the narrative is sufficient for the abstract with the theme numbers more relevant for the body of the paper. Instead the authors could state that there were four global themes identified, two that reflect barriers and two that reflect enablers.**

RESPONSE: We have now deleted the labels ‘Enabler Theme 1’, ‘Barrier Theme 1’, ‘Enabler Theme 2’ and ‘Barrier Theme 2’ from the abstract, and have added the suggested sentence *“Four global themes were identified; two reflecting enablers and two reflecting barriers”.*

**[2] The authors might consider these papers that also looked at barriers and enablers to conducting preclinical animal research:** [**https://can01.safelinks.protection.outlook.com/?url=https%3A%2F%2Fosf.io%2Fwvn85%2F&amp;data=05%7C01%7Cmadfoster%40ohri.ca%7C57133d1ba2474d90a65d08da78a4f048%7C859b41b6130f4d13a6931ffec4e7cb5a%7C0%7C0%7C637954947049750954%7CUnknown%7CTWFpbGZsb3d8eyJWIjoiMC4wLjAwMDAiLCJQIjoiV2luMzIiLCJBTiI6Ik1haWwiLCJXVCI6Mn0%3D%7C3000%7C%7C%7C&amp;sdata=Rtk0jZvKCJE71i2wqUF%2BNUisMZASETLtqOu9e6vY%2FGM%3D&amp;reserved=0**](https://can01.safelinks.protection.outlook.com/?url=https%3A%2F%2Fosf.io%2Fwvn85%2F&amp;data=05%7C01%7Cmadfoster%40ohri.ca%7C57133d1ba2474d90a65d08da78a4f048%7C859b41b6130f4d13a6931ffec4e7cb5a%7C0%7C0%7C637954947049750954%7CUnknown%7CTWFpbGZsb3d8eyJWIjoiMC4wLjAwMDAiLCJQIjoiV2luMzIiLCJBTiI6Ik1haWwiLCJXVCI6Mn0%3D%7C3000%7C%7C%7C&amp;sdata=Rtk0jZvKCJE71i2wqUF%2BNUisMZASETLtqOu9e6vY%2FGM%3D&amp;reserved=0)**&** [**https://can01.safelinks.protection.outlook.com/?url=https%3A%2F%2Fjournals.plos.org%2Fplosone%2Farticle%3Fid%3D10.1371%2Fjournal.pone.0226443&amp;data=05%7C01%7Cmadfoster%40ohri.ca%7C57133d1ba2474d90a65d08da78a4f048%7C859b41b6130f4d13a6931ffec4e7cb5a%7C0%7C0%7C637954947049750954%7CUnknown%7CTWFpbGZsb3d8eyJWIjoiMC4wLjAwMDAiLCJQIjoiV2luMzIiLCJBTiI6Ik1haWwiLCJXVCI6Mn0%3D%7C3000%7C%7C%7C&amp;sdata=xz6b3BzMDiakZB98q%2BthPNM86yD2X%2F%2FjlFskcmUeKiY%3D&amp;reserved=0**](https://can01.safelinks.protection.outlook.com/?url=https%3A%2F%2Fjournals.plos.org%2Fplosone%2Farticle%3Fid%3D10.1371%2Fjournal.pone.0226443&amp;data=05%7C01%7Cmadfoster%40ohri.ca%7C57133d1ba2474d90a65d08da78a4f048%7C859b41b6130f4d13a6931ffec4e7cb5a%7C0%7C0%7C637954947049750954%7CUnknown%7CTWFpbGZsb3d8eyJWIjoiMC4wLjAwMDAiLCJQIjoiV2luMzIiLCJBTiI6Ik1haWwiLCJXVCI6Mn0%3D%7C3000%7C%7C%7C&amp;sdata=xz6b3BzMDiakZB98q%2BthPNM86yD2X%2F%2FjlFskcmUeKiY%3D&amp;reserved=0)

RESPONSE: Thank you for this suggestion – we have now incorporated these references on page 17/18.

**[3] The authors might consider this paper on supporting rigor champions (**[**https://can01.safelinks.protection.outlook.com/?url=https%3A%2F%2Felifesciences.org%2Farticles%2F55915&amp;data=05%7C01%7Cmadfoster%40ohri.ca%7C57133d1ba2474d90a65d08da78a4f048%7C859b41b6130f4d13a6931ffec4e7cb5a%7C0%7C0%7C637954947049750954%7CUnknown%7CTWFpbGZsb3d8eyJWIjoiMC4wLjAwMDAiLCJQIjoiV2luMzIiLCJBTiI6Ik1haWwiLCJXVCI6Mn0%3D%7C3000%7C%7C%7C&amp;sdata=C9feJj9eriNNICZ7V%2BRSdDS5t8TgfK4BK2LStbvQTWE%3D&amp;reserved=0**](https://can01.safelinks.protection.outlook.com/?url=https%3A%2F%2Felifesciences.org%2Farticles%2F55915&amp;data=05%7C01%7Cmadfoster%40ohri.ca%7C57133d1ba2474d90a65d08da78a4f048%7C859b41b6130f4d13a6931ffec4e7cb5a%7C0%7C0%7C637954947049750954%7CUnknown%7CTWFpbGZsb3d8eyJWIjoiMC4wLjAwMDAiLCJQIjoiV2luMzIiLCJBTiI6Ik1haWwiLCJXVCI6Mn0%3D%7C3000%7C%7C%7C&amp;sdata=C9feJj9eriNNICZ7V%2BRSdDS5t8TgfK4BK2LStbvQTWE%3D&amp;reserved=0)**).**

RESPONSE: Thank you, we have now incorporated this as a citation on page 17.

**[4] Related to the above paper and the topic in the paper of enabling rigor champions within the lab, the authors might consider the relevance of this papers' approach to shifting the focus from educating individuals to changing the culture within the lab:** [**https://can01.safelinks.protection.outlook.com/?url=https%3A%2F%2Fwww.pnas.org%2Fdoi%2F10.1073%2Fpnas.1917848117&amp;data=05%7C01%7Cmadfoster%40ohri.ca%7C57133d1ba2474d90a65d08da78a4f048%7C859b41b6130f4d13a6931ffec4e7cb5a%7C0%7C0%7C637954947049750954%7CUnknown%7CTWFpbGZsb3d8eyJWIjoiMC4wLjAwMDAiLCJQIjoiV2luMzIiLCJBTiI6Ik1haWwiLCJXVCI6Mn0%3D%7C3000%7C%7C%7C&amp;sdata=9TCbFvXBxU6J8P9akis6ct2ouuESirFGcH5ZGDVWtjo%3D&amp;reserved=0**](https://can01.safelinks.protection.outlook.com/?url=https%3A%2F%2Fwww.pnas.org%2Fdoi%2F10.1073%2Fpnas.1917848117&amp;data=05%7C01%7Cmadfoster%40ohri.ca%7C57133d1ba2474d90a65d08da78a4f048%7C859b41b6130f4d13a6931ffec4e7cb5a%7C0%7C0%7C637954947049750954%7CUnknown%7CTWFpbGZsb3d8eyJWIjoiMC4wLjAwMDAiLCJQIjoiV2luMzIiLCJBTiI6Ik1haWwiLCJXVCI6Mn0%3D%7C3000%7C%7C%7C&amp;sdata=9TCbFvXBxU6J8P9akis6ct2ouuESirFGcH5ZGDVWtjo%3D&amp;reserved=0)

RESPONSE: Thank you, we have now incorporated this as a citation on page 17.

**[5] The authors posted their protocol file to OSF on July 11, 2018. The file says the start date was May 8, 2018 and completion October 21, 2018 - so does this mean the study protocol was posted half-way through the data collection? As far as I can tell the authors didn't preregister (e.g., it is not available here:** [**https://can01.safelinks.protection.outlook.com/?url=https%3A%2F%2Fosf.io%2Fregistries&amp;data=05%7C01%7Cmadfoster%40ohri.ca%7C57133d1ba2474d90a65d08da78a4f048%7C859b41b6130f4d13a6931ffec4e7cb5a%7C0%7C0%7C637954947049750954%7CUnknown%7CTWFpbGZsb3d8eyJWIjoiMC4wLjAwMDAiLCJQIjoiV2luMzIiLCJBTiI6Ik1haWwiLCJXVCI6Mn0%3D%7C3000%7C%7C%7C&amp;sdata=IOo4X7kxSLhiYAK9peOFFOrZeQrctou58%2BJXJ6RebnI%3D&amp;reserved=0)**](https://can01.safelinks.protection.outlook.com/?url=https%3A%2F%2Fosf.io%2Fregistries&amp;data=05%7C01%7Cmadfoster%40ohri.ca%7C57133d1ba2474d90a65d08da78a4f048%7C859b41b6130f4d13a6931ffec4e7cb5a%7C0%7C0%7C637954947049750954%7CUnknown%7CTWFpbGZsb3d8eyJWIjoiMC4wLjAwMDAiLCJQIjoiV2luMzIiLCJBTiI6Ik1haWwiLCJXVCI6Mn0%3D%7C3000%7C%7C%7C&amp;sdata=IOo4X7kxSLhiYAK9peOFFOrZeQrctou58%2BJXJ6RebnI%3D&amp;reserved=0))**, so I am going off of log/history of the file. This also suggests the interview data come from 2018 - the authors might consider adding what, if anything, has changed from when these interviews were performed vs now. Also, I suggest indicating 2018 was when the interviews were performed in the papers methods section for increased transparency for the reader.**

RESPONSE: We would note that formal registration of qualitative studies in OSF was only enabled recently with a qualitative specific template. Thus, we posted the protocol in a time-stamped manner. The posted protocol included the start and anticipated completion date (May 8 – Oct 31, 2018). Interviews were conducted from May 16, 2018- January 8, 2019. Our initial protocol was submitted and approved by the Research Ethics Board (REB) prior to the start of the study. There was a delay in posting to OSF to allow for incorporation of details that were captured within the main REB application platform and incorporation of an amendment that was approved by the Research Ethics Board (gift card for participants). We would also note that the protocol was posted well before any analysis was conducted.

We have now made the timing of interviews more transparent in the Methods section:

*“Participants provided written informed consent and were subsequently interviewed in person or by telephone between May 16, 2018 to January 8, 2019, by a trained research assistant with extensive interview experience and a background in health science and laboratory research (MF).”*

**[6] Related to the point above, in methods the authors state their sampling strategy was pre-determined, yet there is no indication of the approach in the OSF file.**

RESPONSE: The sampling strategy was detailed under the “Recruitment Plan” section of the posted protocol. In terms of the sample size, for completeness we should have mentioned the 10+3 rule; however, we would note this is a rule that we have followed for all other TDF based interview studies by our group.
