# Supplementary material for: Identifying barriers and enablers to rigorous conduct and reporting of preclinical laboratory studies
Source: PLoS Biol. 2023 Jan 5;21(1):e3001932. doi: 10.1371/journal.pbio.3001932 (PMC9888705; doi:10.1371/journal.pbio.3001932)
Supplement: Attachment — Submitted filename: Response to Editor Reviews_Nov 16.docx [file pbio.3001932.s012.docx]

Dear PLOS Biology Editors & Academic Editor,

Thank you for your review of our revised manuscript "Identifying barriers and enablers to rigorous conduct and reporting of preclinical studies" for publication as a Meta-Research Article at PLOS Biology. Below, please find our responses outlining changes made to address the proposed suggestions. We feel these suggestions have helped to strengthen the transparency of our study.

a) Competing interests - you currently list the following competing interest: "MML has previously acted as a guest editor for PLOS Biology." We would not normally consider this as a competing interest, but are fine if you want to leave it as-is.

Thank you for raising this – we have removed this from the competing interest section; our updated response now reads: “The authors have declared that no competing interests exist.”

b) Ethics - You say that the study "complies with all relevant ethical regulations." but do not specify which national/international guidelines the study adheres to (e.g. Declaration of Helsinki) - please could you clarify this in the text?

Thank you for raising this important point. We have now adjusted so that the Ethics statement reads:

“This study was approved through the Ottawa Health Science Network Research Ethics Board (Protocol 20170659-01H) and complies with the Canadian Tri-Council Policy Statement: Ethical Conduct for Research Involving Humans.”

c) Data availability - we agree that we should not insist on you providing the full transcripts, as requested by R1, for the reasons that you mention in your rebuttal. However, Table 1 has some values for which you only present median and range values (age of participants, experience, length of interview); please could you supply the underlying numerical values for these as a supplementary data file, S1_Data.xslx?

We have now included the file S1 Data, which outlines the underlying numerical values for experience and length of interview. We unfortunately are not able to disclose the numerical data for gender and age as our ethics application had specified that these individual participant data would not be shared for these variables.

d) Please cite the location of the data clearly in the legend to Table 1, e.g. “The data underlying this Figure can be found in S1 Data”.

We have now added the below sentence to the legend of Table 1:

“The data underlying this Figure can be found in S1 Data.”
